# Self-rated health and risk of incident non-alcoholic fatty liver disease: A cohort study

**DOI:** 10.1038/s41598-020-60823-8

**Published:** 2020-03-02

**Authors:** Yoosoo Chang, Jin-Won Noh, Joo Young Cheon, Yejin Kim, Young Dae Kwon, Seungho Ryu

**Affiliations:** 1 Department of Occupational and Environmental Medicine, Kangbuk Samsung Hospital, Sungkyunkwan University School of Medicine, Seoul, Republic of Korea; 2 Center for Cohort Studies, Total Healthcare Center, Kangbuk Samsung Hospital, Sungkyunkwan University School of Medicine Seoul, Seoul, Republic of Korea; 3 Department of Clinical Research Design & Evaluation, SAIHST, Sungkyunkwan University, Seoul, Republic of Korea; 4 Department of Health Administration, College of Health Science, Dankook University, Cheonan, Republic of Korea; 5 Institute of Health Promotion and Policy, Dankook University, Cheonan, Republic of Korea; 6 Global Health Unit, Department of Health Sciences, University Medical Center Groningen, University of Groningen, Groningen, the Netherlands; 7 Department of Nursing Science, Sungshin University, Seoul, Republic of Korea; 8 Department of Humanities and Social Medicine, College of Medicine and Catholic Institute for Healthcare Management, The Catholic University of Korea, Seoul, Republic of Korea

**Keywords:** Non-alcoholic fatty liver disease, Risk factors

## Abstract

Although self-rated health (SRH), a subjective measure of overall health status, associates with metabolic abnormalities, studies on the relationship between SRH and non-alcoholic fatty liver disease (NAFLD), a hepatic manifestation of metabolic syndrome, are limited. In this study, we evaluated whether or not SRH predicts the risk of incident NAFLD. This cohort study was performed in a sample of 148,313 Korean adults free of ultrasound-diagnosed NAFLD at baseline with annual or biennial follow-up for a median of 3.7 years. SRH and NAFLD were measured at baseline and follow-up visits. NAFLD was determined based on the ultrasound-diagnosed fatty liver without excessive alcohol consumption or any other cause. Hazard ratios with 95% confidence intervals were estimated via a parametric proportional hazards model. During 522,696.1 person-years of follow-up, 23,855 individuals with new-onset NAFLD were identified (incidence rate, 45.6 per 1,000 person-years). After adjustments for possible confounders including total calorie intake, sleep duration, and depressive symptoms, the multivariate-adjusted hazard ratios (95% confidence intervals) for incident NAFLD comparing good, fair, and poor or very poor SRH to very good SRH were 1.06 (0.97–1.14), 1.18 (1.09–1.27), and 1.24 (1.13–1.37), respectively. This association of SRH with incident NAFLD remained significant after accounting for changes in SRH and confounders during follow-up and was similar across clinically relevant subgroups. In a large-scale cohort study of apparently healthy Korean adults, poor SRH was independently and positively associated with incident NAFLD risk, indicating a predictive role of SRH as a health measure in NAFLD.

## Introduction

Nonalcoholic fatty liver disease (NAFLD) is the most common liver disease globally and encompasses the bland steatosis to nonalcoholic steatohepatitis (NASH) that has potential to develop into progressive liver disease including cirrhosis, hepatic failure, or liver cancer^1,2^. In addition to liver-related adverse outcomes^3^, NAFLD is also associated with metabolic abnormalities, renal disorders and cerebrovascular or cardiovascular disease (CVD)^4–6^ and is considered a hepatic manifestation of metabolic syndrome (MetS) as well as a multisystem disease^7–9^. Given the increasing burden of NAFLD and its liver and extrahepatic complications, it is crucial to develop preventive measures for targeting individuals at risk for NAFLD.

Self-rated health (SRH) is a reliable indicator to assess personal perceptions of subjective overall health status by a single question and is widely used in various health research fields^10^. Indeed, SRH has been reported to be independently predictive of various adverse health outcomes such as CVD, cerebrovascular disease, respiratory disease, functional deterioration, depressive symptoms, and all-cause mortality^11^. SRH is likely to capture the net effects of various measured and unmeasured risk factors^12^. Previous studies demonstrated relationships of poor SRH with unhealthy lifestyle^13–15^ and metabolic derangements such as obesity, diabetes, MetS, and metabolically unhealthy status, all of which are closely related to insulin resistance and are also risk factors of NAFLD^16–18^. Additionally, poor SRH has been associated with elevated inflammatory markers and insulin resistance, important key factors for NAFLD^19,20^. Until now, there are few studies to evaluate whether or not SRH predicts NAFLD.

Thus, we investigated the longitudinal relationship between SRH and new-onset NAFLD in a large cohort of apparently healthy Korean adults who underwent a health check-up examination with repeated measurements of SRH, NAFLD and other confounders including lifestyle habits, comorbidities, and metabolic parameters.

## Materials and Methods

### Study population

The present study is part of the Kangbuk Samsung Health Study, a cohort study of Korean men and women aged 18 years or older who annually or biannually underwent comprehensive health examinations at the Kangbuk Samsung Hospital Health Screening Centers in Seoul or Suwon, Republic of Korea^21^. Most participants were employees of companies and local governmental organizations and their spouses. In Korea, annual or biennial health screening exams of the employees are required by the Industrial Safety and Health Law. The rest of the examinees voluntarily underwent self-paid health checkups.

**Figure 1:**
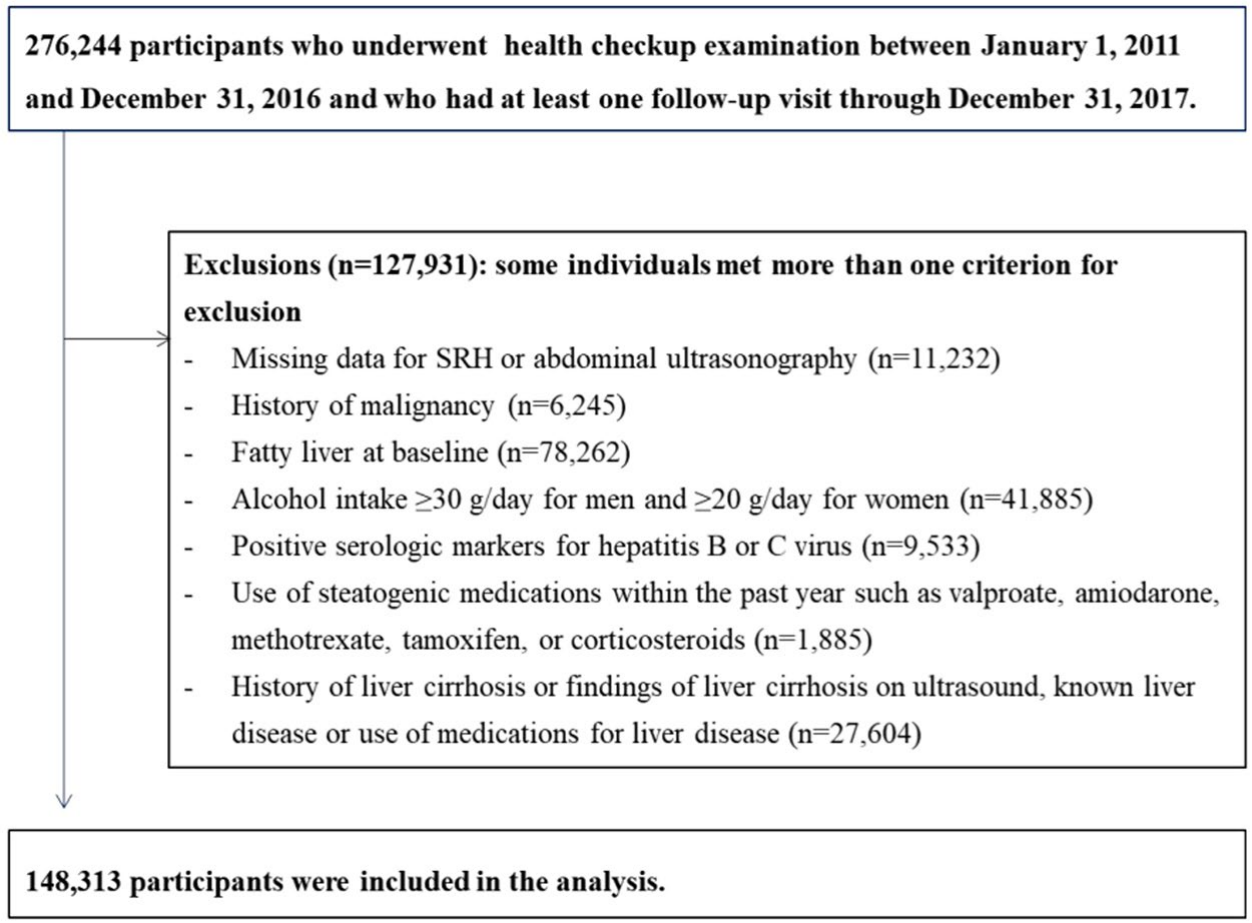
Selection of the study population.

This study involved examinees who underwent comprehensive examination from 2011 to 2016 and who participated in at least one follow-up examination until the end of 2017 (n = 276,244). Participants were excluded for the following reasons at baseline: missing information on SRH or abdominal ultrasonography (n = 11,232); history of cancer (n = 6,245); type 2 diabetes (n = 10,170); fatty liver on ultrasound (n = 78,262); significant alcohol consumption (defined as alcohol intake ≥30 g/day for men and ≥20 g/day for women) (n = 41,885)^2^; positive serologic markers for hepatitis B or C virus (n = 9,533); use of steatogenic medications within the past year such as valproate, amiodarone, methotrexate, tamoxifen, or corticosteroids (n = 1,885)^2^; history of liver cirrhosis or ultrasound-diagnosed liver cirrhosis, or any type of liver disease (n = 27,604). Some individuals met more than one exclusion criterion, yielding a final analytic sample of 148,313 (Fig. 1).

The study was approved by the Institutional Review Board of Kangbuk Samsung Hospital, which waived the requirement for informed consent as we used only de-identified data routinely collected as part of a health screening program.

### Measurements

Abdominal ultrasonography, blood tests and physical parameters were routinely measured as part of the basic health check-up program at baseline and follow-up visits. Data on demographic factors, socioeconomic status, health behaviors, medical history and medication use were collected by standardized, structured, self-administered questionnaires^22^. Health behaviors and socioeconomic status were categorized as follows: smoking status (never, former, or current smoker), alcohol consumption (≤10 g/day and >10 g/day), physical activity (inactive, minimally active and health-enhancing physical activity (HEPA)), sleep duration (≤5, 6, 7, 8, or ≥9 hour), monthly household income (<6 million Korean won (4907 USD) or ≥6 million Korean won), job status (full-time job or part-time job) and education attainment (less than college graduate or college graduate or more). HEPA was defined when either of two criteria were satisfied: (i) vigorous-intensity activity on ≥3 days per week accumulating ≥1,500 metabolic equivalent task (MET) min/week; or (ii) seven days of any combination of walking, moderate-intensity, or vigorous-intensity activities achieving at least 3,000 MET min/week^23^. Usual dietary intake was assessed via a 106-item, semi-quantitative, self-administered food frequency questionnaire for Korean^24^. Depressive symptoms were assessed using the Korean version^25^ of the Center for Epidemiologic Studies Depression (CES-D) Scale^26^ and were categorized as CES-D score <16, 16–24, and ≥25.

SRH was assessed using a self-administered questionnaire in Korean at baseline and follow-up visits and was defined by responses to a single question such as “In general, how would you rate your health?”, with the possible choices of “very good” (1), “good” (2), “fair” (3), “poor” (4) or “very poor” (5)^27,28^. There have been a number of studies investigating the content validity of SRH across different ethnicities and populations^27,29^.

Anthropometric parameters were measured by trained nurses. Obesity was defined as body mass index (BMI) ≥25 kg/m^2^ according to criteria for obesity in Asians^30^. Blood pressures (BP) were measured using an automated oscillometric device (53000, Welch Allyn, NY, USA) by trained nurses while examinees were sitting with the arm supported at heart level. Hypertension was defined as BP ≥140/90 mmHg or current use of BP-lowering agents. Type 2 diabetes mellitus was defined as fasting serum glucose ≥126 mg/dL, hemoglobin A1c (HbA1c) ≥6.5%, or current use of insulin or medications for diabetes.

Fasting blood measurements included glucose, HbA1c, alanine aminotransferase (ALT), insulin, high sensitivity C-reactive protein (hsCRP), and lipid profiles including total cholesterol, low-density lipoprotein cholesterol (LDL-C), high-density lipoprotein cholesterol (HDL-C), and triglycerides. The homeostatic model assessment – insulin resistance (HOMA-IR) was calculated as follows: fasting blood insulin (uU/ml) × fasting blood glucose (mg/dL)/405.

Fatty liver was assessed based on abdominal ultrasound by experienced radiologists who were not aware of the study purpose with participants in the supine position with the right arm raised above the head.^31^ Ultrasonographic diagnosis of fatty liver was based on standard criteria, including diffuse increase of fine echoes in the liver parenchyma compared with the kidney or spleen parenchyma, deep beam attenuation, and bright vessel walls.^32^ The inter- and intra-observer reliability for diagnosis of fatty liver was very high (kappa statistics of 0.74 and 0.94, respectively)^33^. Since we had excluded participants with significant alcohol consumption and other causes of hepatic steatosis at baseline (see further details in the exclusion criteria), incident cases of fatty liver were considered NAFLD.

### Statistical analyses

SRH was categorized into four groups: (1) very good, (2) good, (3) fair, or 4) poor or very poor. Only a small proportion of participants responded very poor health, which was combined into the poor category. Characteristics of the study participants were presented by SRH. The development of NAFLD was the primary outcome. The incidence rate was expressed as the number of new-onset NAFLD cases per 1000 person-years. Since we knew that NAFLD had occurred at some point between the two visits but did not know the precise timing of NAFLD development, we used a parametric proportional hazards model to account for this type of interval censoring (*stpm* command in Stata)^34^.

The adjusted hazard ratio (aHR) and 95% confidence interval (CI) for incident NAFLD were estimated using a parametric proportional hazards model. Models were initially adjusted for age and sex and then further adjusted for year of screening examination, center, education attainment, monthly household income, employment status, smoking status, alcohol consumption, physical activity, sleep duration, CES-D, total calorie intake, BMI, history of CVD, and history of hypertension (multivariable-adjusted model). To incorporate the change of SRH and confounders during follow-up, we performed time-dependent analyses while introducing SRH and other covariates as a time-varying covariate in the models. We assessed the proportional hazards assumption by examining graphs of estimated log (−log) survival; no violation of the assumption was found.

In addition, we performed subgroup analyses by age (<50 vs. ≥50 years), sex (men vs. women), current smoking (yes vs. no), alcohol consumption (<10 vs. ≥10 g/day), HEPA (no vs. yes), and BMI (<25 vs. ≥25 kg/m^2^). Likelihood ratio tests comparing models with and without multiplicative interaction terms were used to evaluate interactions by subgroup characteristics. Statistical analyses were performed using STATA version 15.0 (StataCorp LP, College Station, TX, USA). All *p*-values were two-tailed, and *p*-values < 0.05 were considered statistically significant.

## Results

The average (SD) age of participants was 38.3 (7.7) years (Table 1). Age, male sex, high education attainment, high monthly household income, and alcohol consumption were positively associated with better SRH category, whereas part-time job (vs. full-time job), hypertension, a history of CVD, depressive symptoms based on CES-D, insulin resistance estimated by HOMA-IR, and systemic inflammation measured by hsCRP were associated with poorer SRH category.

**Table 1:** Baseline characteristics of study participants by self-rated health.

Characteristics	Overall	Self-rated health category				*P* value	Multiple comparison
		Very good (a)	Good (b)	Fair (c)	Poor or very poor (d)		
Number	148,313	4,918	46,617	86,617	10,091		
Age (years)^a^	37.4 (7.5)	38.1 (8.5)	37.4 (7.6)	37.4 (7.4)	36.9 (7.7)	<0.001	a≠b, a≠c, a≠d, b≠d, c≠d
Male (%)	38.9	53.3	46.4	35.2	29.3	<0.001	a≠b≠c≠d
Current smoker (%)	18.5	18.6	19.4	18.1	18.1	<0.001	b≠c, b≠d
Alcohol intake (%)^b^	25.6	29.1	28.2	24.6	21.3	<0.001	a≠c, a≠d, b≠c, b≠d, c≠d
HEPA (%)	15.4	31.0	19.9	12.7	10.2	<0.001	a≠b≠c≠d
High education level (%)^c^	84.3	85.8	86.5	83.5	80.6	<0.001	a≠c, a≠d, b≠c, b≠d, c≠d
High income (%)^d^	32.4	36.7	34.6	31.3	29.2	<0.001	a≠b≠c≠d
Part time job (%)	7.6	6.1	6.7	8.2	8.3	<0.001	a≠c, a≠d, b≠c, b≠d
History of CVD (%)	0.6	0.5	0.5	0.7	1.2	<0.001	a≠d, b≠c, b≠d, c≠d
Hypertension (%)	5.1	4.6	4.60	5.2	6.1	<0.001	a≠d, b≠c, b≠d, c≠d
Sleep duration (hours)	7.0 (6.0–7.0)	7.0 (6.0–7.0)	7.0 (6.0–7.0)	7.0 (6.0–7.0)	6.0 (6.0–7.0)	<0.001	a≠d, b≠d, c≠d, b≠c
CES–D ≥16 (%)	11.9	3.6	6.0	13.1	33.1	<0.001	a≠b≠c≠d
Obesity (%)	11.8	13.9	12.2	11.4	12.0	<0.001	a≠b, a≠c, a≠d, b≠c
BMI (kg/m^2^)	21.9 (2.6)	22.4 (2.4)	22.1 (2.5)	21.7 (2.7)	21.6 (3.0)	<0.001	a≠b≠c≠d
Systolic BP (mmHg)^a^	105.4 (12.0)	106.9 (12.0)	106.2 (12.0)	105.1 (12.1)	103.7 (11.7)	<0.001	a≠b≠c≠d
Diastolic BP (mmHg)^a^	67.2 (9.0)	67.7 (8.9)	67.5 (8.9)	67.1 (9.0)	66.4 (8.7)	<0.001	a≠c, a≠d, b≠c, b≠d, c≠d
Glucose (mg/dl)^a^	91.6 (7.8)	91.7 (8.0)	91.8 (7.9)	91.6 (7.8)	91.1 (7.8)	<0.001	a≠d, b≠c, b≠d, b≠d
Total cholesterol (mg/dl)^a^	187.6 (31.6)	187.7 (30.9)	187.9 (31.2)	187.7 (31.8)	185.8 (32.2)	<0.001	a≠d, b≠d, b≠d,
LDL-C (mg/dl)^a^	113.0 (29.2)	112.9 (28.7)	113.3 (28.8)	113.0 (29.4)	111.4 (29.5)	<0.001	a≠d, b≠d, b≠d,
HDL-C (mg/dl)^a^	62.6 (14.6)	63.1 (14.9)	62.8 (14.6)	62.6 (14.6)	62.3 (14.4)	0.001	a≠d
Triglycerides (mg/dl)^e^	75 (57–103)	74 (56–101)	75 (57–102)	75 (57–103)	75 (57–102)	0.035	a≠c
ALT (U/l)^e^	15 (11–20)	15 (12–21)	15 (11–20)	14 (11–20)	14 (11–19)	<0.001	a≠b, a≠c, a≠d, b≠c, b≠d
HOMA-IR^e^	1.00 (0.67–1.43)	0.93 (0.63–1.34)	0.97 (0.66–1.38)	1.02 (0.69–1.45)	1.03 (0.68–1.49)	<0.001	a≠c, a≠d, b≠c, b≠d, c≠d
hsCRP (mg/l)^e^	0.3 (0.2–0.6)	0.3 (0.2–0.6)	0.3 (0.2–0.6)	0.3 (0.2–0.6)	0.3 (0.2–0.7)	<0.001	a≠b, a≠c, a≠d, b≠c, b≠d
Total energy intake (kcal/day)^e,f^	1509.9 (1158.0–1894.0)	1549.9 (1170.2–1988.0)	1539.2 (1187.1–1924.5)	1493.5 (1146.8–1872.0)	1501.3 (1142.1–1889.1)	<0.001	a≠c, a≠d, b≠c, b≠d,

Data are ^a^mean (standard deviation), ^e^median (interquartile range), or percentage.

Abbreviations: ALT, alanine aminotransferase; BMI, body mass index; BP, blood pressure; CES-D, Center for Epidemiologic Studies Depression; CVD, cardiovascular disease; HDL-C, high-density lipoprotein-cholesterol; HEPA, health-enhancing physical activity; HOMA-IR, homeostasis model assessment of insulin resistance; hsCRP, high sensitivity C-reactive protein; LDL-C, low-density lipoprotein cholesterol.

^b^≥ 10 g of ethanol per day ^c^≥ college graduate ^d^monthly household income ≥6 million Korean won. ^f^among 175,345 participants with plausible estimated energy intake levels (within three standard deviations from the log-transformed mean energy intake).

During a median follow-up period of 3.7 years (interquartile range, 2.0–4.9 years; 522,696.1 person-years of follow-up), 23,855 new cases of NAFLD were identified, with a corresponding incidence rate of 45.6 per 1,000 person-years. Poorer SRH was associated with a higher risk of developing NAFLD (Table 2). The age- and sex-adjusted HRs (95% CIs) for incident NAFLD comparing the good, fair, and poor or very poor vs. the very good SRH categories were 1.03 (0.96–1.11), 1.13 (1.05–1.21), and 1.27 (1.17–1.38), respectively (*P* for trend <0.001). After further adjustments for possible confounders including socioeconomic status (monthly household income, job status, education attainment), health behaviors, sleep duration, and depressive symptoms, the multivariable-adjusted hazard ratios (95% CIs) for NAFLD in good, fair, and poor or very poor SRH categories were 1.06 (0.97–1.14), 1.18 (1.09–1.27), and 1.24 (1.13–1.37), respectively, compared with the very good SRH category (multivariable-adjusted model). Further adjustment for either HOMA-IR, hsCRP, or lipid profiles including LDL-C, HDL-C, and triglycerides slightly attenuated the association between SRH and incident NAFLD but did not eliminate it (data not shown). When updated status in SRH and confounders over follow-up were treated as time-varying covariates, SRH independently associated with incident NAFLD in time-dependent models.

**Table 2:** Development of nonalcoholic fatty liver disease according to self-rated health category.

Self-rated health category	Person-years (PY)	Incident case	Incidence density (per 10^3^ PY)	Age and sex-adjusted HR (95% CI)	Multivariable-adjusted HR^a^ (95% CI)	HR (95% CI)^b^ in model using time-dependent variables
Very good	16,711.6	812	48.6	1.00 (reference)	1.00 (reference)	1.00 (reference)
Good	165,572.6	7,759	46.9	1.03 (0.96–1.11)	1.06 (0.97–1.14)	1.04 (0.96–1.12)
Fair	305,328.9	13,688	44.8	1.13 (1.05–1.21)	1.18 (1.09–1.27)	1.14 (1.05–1.23)
Poor or very poor	35,083.0	1,596	45.5	1.27 (1.17–1.38)	1.24 (1.13–1.37)	1.21 (1.10–1.33)
P for trend				<0.001	<0.001	<0.001

^a^Estimated from parametric proportional hazard models. Multivariable model was adjusted for age, center, year of screening exam, smoking status, alcohol intake, physical activity, education level, total calorie intake, body mass index, sleep duration, CES-D, monthly household income, part-time job, history of hypertension, and history of cardiovascular disease; model 2: model 1 plus adjustment for hsCRP and HOMA-IR.

^b^Estimated from parametric proportional hazard models with self-rated health category, smoking status, alcohol intake, physical activity, BMI, total calorie intake, sleep duration, and CESD category as time-dependent variables and baseline age, sex, center, year of screening exam, education level, history of hypertension and history of CVD as time-fixed variables.

Abbreviations: CES-D, Center for Epidemiologic Studies Depression; CI, confidence interval; HR, hazard ratio.

Association between SRH and incident NAFLD was similar across various subgroups without significant interactions by age (<50 vs. ≥50 years), sex (women vs. men), current smoking (no vs. yes), HEPA (no vs. yes), or obesity (no vs. yes) (Table 3 and Supplementary Table 1).

**Table 3:** Hazard ratios^a^ (95% CI) of incident nonalcoholic fatty liver disease according to self-rated health category in clinically relevant subgroups.

Subgroup	Self-rated health category				*P* for trend	*P* for interaction
	Very good	Good	Fair	Poor or very poor		
Age						0.378
<50 years (N = 138,921)	reference	1.02 (0.94–1.11)	1.14 (1.05–1.24)	1.19 (1.08–1.31)	<0.001	
≥50 years (N = 9,392)	reference	1.27 (0.97–1.65)	1.41 (1.09–1.82)	1.60 (1.15–2.23)	0.001	
Sex						0.465
Women (N = 90,590)	reference	1.10 (0.93–1.31)	1.20 (1.01–1.42)	1.32 (1.10–1.59)	<0.001	
Men (N = 57,723)	reference	1.04 (0.95–1.14)	1.17 (1.07–1.28)	1.19 (1.06–1.34)	<0.001	
Current smoking						0.485
No (N = 106,920)	reference	1.09 (0.98–1.20)	1.18 (1.07–1.31)	1.28 (1.13–1.44)	<0.001	
Yes (N = 24,337)	reference	1.04 (0.89–1.22)	1.20 (1.03–1.40)	1.25 (1.04–1.50)	<0.001	
Alcohol intake						0.084
<10 g/day (N = 102,005)	reference	1.11 (0.99–1.23)	1.20 (1.08–1.33)	1.28 (1.13–1.45)	<0.001	
≥10 g/day (N = 35,176)	reference	1.10 (0.89–1.22)	1.18 (1.04–1.34)	1.20 (1.02–1.41)	<0.001	
HEPA						0.419
No (N = 124,325)	reference	1.06 (0.97–1.17)	1.18 (1.07–1.30)	1.23 (1.10–1.37)	<0.001	
Yes (N = 22,556)	reference	1.03 (0.89–1.19)	1.18 (1.03–1.36)	1.38 (1.12–1.71)	<0.001	
Body mass index						0.133
<25 kg/m^2^ (N = 130,738)	reference	1.11 (1.01–1.22)	1.25 (1.14–1.38)	1.31 (1.16–1.47)	<0.001	
≥25 kg/m^2^ (N = 17,453)	reference	0.94 (0.82–1.09)	1.03 (0.90–1.18)	1.11 (0.94–1.31)	0.001	

^a^Estimated from parametric proportional hazard models adjusted for age, sex, center, year of screening exam, smoking status, alcohol intake, physical activity, education level, total calorie intake, body mass index, sleep duration, CES-D, monthly household income, part-time job, history of hypertension, and history of cardiovascular disease.

Abbreviations: CES-D, Center for Epidemiologic Studies Depression; HEPA, health-enhancing physically active; HOMA-IR, homeostasis model assessment of insulin resistance; hsCRP, high sensitivity C-reactive protein.

## Discussion

In this cohort study of 148,313 Korean adults free of ultrasound-diagnosed NAFLD at baseline, lower SRH was associated with an increased risk of developing NAFLD in a dose-dependent manner. This association was independent of potential confounders, including employment status, education attainment, household income, depressive symptoms, comorbidities, and lifestyle factors and was consistently observed when updated status of SRH and confounders at follow-up visits were incorporated as time-varying covariates. Therefore, the findings of the present study support an independent and predictive role of SRH as a health measure in the development of NAFLD. SRH may help identify people who have a high risk of future NAFLD.

Some studies exploring the relationship between SRH and NAFLD have shown conflicting results^35^. In a cross-sectional study of a nationally representative population in the United States, assessment of SRH was included as a subscale of health-related quality of life through the question, “Rate your health status as poor, fair, good, very good or excellent”^35^. In that study, NAFLD was associated with poorer SRH^35^. However, SRH was compared among three groups (the group with chronic hepatitis C, the group with NAFLD, and healthy control group) without adjusting for lifestyle factors, depression, or laboratory testing, which are considered important confounders^36,37^. Therefore, it is unclear whether or not that observed association between SRH and NAFLD is independent due to lack of adjustment of potential confounders and it is also limited by their temporal ambiguity between SRH and NAFLD. Another study examined the relationship between NAFLD and self-reported general health perception in a sub-sample (213 participants) of the Israel national health and nutrition examination survey^38^. That study showed an inverse association between NAFLD and the prevalence of “very good” self-reported health perception, but this association was no longer observed after adjustment for BMI. Furthermore, in that study, the presence of NAFLD compared to the normal liver at the baseline survey did not predict perceptions of health deterioration after seven years of follow-up^38^. However, contrary to our study, that study focused on the impact of NAFLD on health perception while treating NAFLD as a predictive variable and SRH as a primary outcome. Before the present study, no cohort studies had examined the prospective association between SRH and new-onset of NAFLD. This is the first cohort study to show a positive and independent association between poorer SRH and incident NAFLD after adjusting for possible subjective and objective confounding factors such as demographic factors, lifestyle behaviors, depressive symptoms, comorbidities, insulin resistance, and systemic inflammation among 148,313 young and middle-aged adults without ultrasound-diagnosed NAFLD at baseline using a prospective cohort study design and repeated measurements including SRH, fatty liver status, and other covariates.

The mechanisms underlying the association between SRH and the development of NAFLD are not fully elucidated yet. NAFLD is attributed to unhealthy health behaviors that are closely associated with excessive adiposity, unfavorable lipid profiles and insulin resistance^39–41^. Furthermore, SRH has been reported to be associated with behavioral factors such as physical activity, smoking, dietary habit, alcohol drinking, and excessive adiposity, all of which are closely correlated with NAFLD^13–15^. SRH may comprehensively reflect lifestyle-related health conditions^42^. However, the relationship of poorer SRH with NAFLD risk, in our study, was observed even after adjustments for various lifestyle factors as well as depressive symptoms. Poor SRH has been reported to be associated with elevated inflammatory markers, another important key factor for NAFLD^19,20^. Thus, poor SRH might reflect the inflammatory milieu in favor of development of NAFLD and be related to symptoms related to systemic inflammation such as fatigue, malaise, sleepiness and reduced energy availability, which might prevent individuals from engaging in physical activity or adopting healthy lifestyles and affect perceived physical functioning, one of the subscales of SRH^43^. However, in our study, further adjustment for lifestyle factors, hsCRP, and HOMA-IR did not fully attenuate the association between SRH and incident NAFLD. Even though SRH is easily assessed through a single-item question about “general health,” this question can be used to assess different dimensions of individual health status according to subjects’ own definition of health, which may include overall subjective and objective health at that point, possibly capturing an overall picture of individual’s health beyond the conventionally measured health conditions. Indeed, the pathogenesis of NAFLD is not clear and seems to be multifactorial with multiple proposed mechanisms^44^. Our study findings support SRH as a predictor of incident NAFLD independent of known NAFLD risk factors to help identify individuals at high risk for NAFLD, with the goal of implementing strategies to prevent incident NAFLD. However, further studies are required to determine what aspects of health experience can affect self-rated health, to determine whether sub-scales of SRH provide a sensitive marker of NAFLD risk, and to clarify whether the use of SRH as a part of actual consultation in clinical settings can improve patient health outcomes including NAFLD.

We note several limitations of the present study that are important to consider during the interpretation of the findings. First, we used ultrasonography to diagnose NAFLD without confirmation by liver biopsy, a gold standard for diagnosis of NAFLD. However, liver biopsy was neither feasible nor ethical in our large, low-risk individuals due to its invasive nature. Although abdominal ultrasound is widely used in population-based epidemiological studies with an acceptable diagnostic accuracy for diagnosing hepatic steatosis^45^, it cannot reliably detect fatty infiltration of the liver below the threshold of 20 to 33%^46,47^. Second, health behaviors were assessed based on self-administered questionnaires; thus, some degree of residual confounding might have been introduced by social desirability bias or measurement errors. Third, anxiety has been linked to poorer SRH as well as subjective well-being in other population-based studies^48–50^. However, information on anxiety symptoms, in our study, was not available until 2017, limiting our ability to consider it in the analysis. Thus, we cannot exclude the possibility of residual confounding related to measurement errors and unmeasured confounders that might explain the observed association between SRH and incident NAFLD. Finally, our study population is much younger than those in previous studies on SRH: only 6.3% of the study population was 50 years old or older. Even though the interaction by age on the association between SRH and incident NAFLD was not statistically significant in our study, the association between SRH and incident NAFLD tended to be stronger in those 50 years or older (vs. younger group). Previous studies have shown that the role or meaning of SRH in predicting health outcomes might differ by age group^51,52^. Therefore, study findings derived from young and middle-aged Koreans who attended health-screening examinations might not be generalizable to other populations with different characteristics such as age, race/ethnicity, or socioeconomic status. On the other hand, the relatively young age of our cohort participants may be a strength because our results have implications in young adults with poor SRH for future risk of NAFLD in relatively short-term follow-up and long-standing NAFLD since young adulthood, which might adversely health outcomes such as liver- and non-liver complications.

In conclusion, individuals with poorer SRH had a higher risk of new-onset NAFLD, indicating that SRH is useful as a predictive health measure for various adverse health outcomes including NAFLD. Health care providers should consider the predictive role of SRH in incident NAFLD among preventive strategies for NAFLD. Further prospective studies are warranted to better understand the mechanism by how SRH predicts incident NAFLD.

## Supplementary information

Supplementary Table 1.
