# Supplementary material for: Self-rated health and risk of incident non-alcoholic fatty liver disease: A cohort study
Source: Sci Rep. 2020 Mar 2;10:3826. doi: 10.1038/s41598-020-60823-8 (PMC7052149; doi:10.1038/s41598-020-60823-8)
Supplement: Supplementary file 1 — Supplementary Table 1. [file 41598_2020_60823_MOESM1_ESM.docx]

**Self-rated health and risk of incident non-alcoholic fatty liver disease: A cohort study**

Yoosoo Chang^1,2,3*^, Jin-Won Noh^4,5,6*^, Joo Young Cheon^7^, Yejin Kim^2^, Young Dae Kwon^8^, Seungho Ryu^1,2,3^

*^1^Department of Occupational and Environmental Medicine, Kangbuk Samsung Hospital, Sungkyunkwan University School of Medicine, Seoul, Republic of Korea; ^2^Center for Cohort Studies, Total Healthcare Center, Kangbuk Samsung Hospital, Sungkyunkwan University School of Medicine Seoul, Republic of Korea;^3^Department of Clinical Research Design & Evaluation, SAIHST, Sungkyunkwan University, Seoul, Republic of Korea; ^4^ Department of Health Administration, College of Health Science, Dankook University, Cheonan, Republic of Korea; ^5^Institute of Health Promotion and Policy, Dankook University, Cheonan, Republic of Korea;^6^Global Health Unit, Department of Health Sciences, University Medical Center Groningen, University of Groningen, Groningen, the Netherlands; ^7^Department of Nursing Science, Sungshin University, Seoul, Republic of Korea;* ^8^*Department of Humanities and Social Medicine, College of Medicine and Catholic Institute for Healthcare Management, The Catholic University of Korea, Seoul, Republic of Korea*

**^*^Yoosoo Chang and Jin-Won Noh contributed equally as co-first authors.**

**Running title:** Self-rated health and fatty liver

**Address for correspondence:** Seungho Ryu, MD, PhD, Kangbuk Samsung Hospital, Samsung Main Building B2, 250, Taepyung-ro 2ga, Jung-gu, Seoul, South Korea 04514

E-mail: [sh703.yoo@gmail.com](mailto:sh703.yoo@gmail.com). Telephone: 82-2-2001-5137. Fax: 82-2-757-0436.

Supplemental Table 1. Development of nonalcoholic fatty liver disease (NAFLD) according to self-rated health category

| Self-rated health category | Person-years (PY) | Incident  case | Incidence density (per 10^3^ PY) | Age-adjusted HR (95% CI) | Multivariable adjusted HR^a^ (95% CI) |
| --- | --- | --- | --- | --- | --- |
| Women |  |  |  |  |  |
| Very good | 7,995.9 | 189 | 23.6 | 1.00 (reference) | 1.00 (reference) |
| Good | 90,542.9 | 2,191 | 24.2 | 1.03 (0.88-1.19) | 1.10 (0.93-1.31) |
| Fair | 200,768.2 | 5,206 | 25.9 | 1.10 (0.95-1.28) | 1.20 (1.01-1.42) |
| Poor or very poor | 25,244.8 | 774 | 30.7 | 1.33 (1.13-1.55) | 1.33 (1.10-1.60) |
| P for trend |  |  |  | <0.001 | <0.001 |
| Men |  |  |  |  |  |
| Very good | 8,715.7 | 623 | 71.4 | 1.00 (reference) | 1.00 (reference) |
| Good | 75,029.7 | 5,568 | 74.2 | 1.04 (0.95-1.13) | 1.04 (0.95-1.14) |
| Fair | 104,560.7 | 8,482 | 81.1 | 1.14 (1.05-1.24) | 1.17 (1.07-1.28) |
| Poor or very poor | 9,838.3 | 822 | 83.6 | 1.21 (1.09-1.35) | 1.19 (1.06-1.34) |
| P for trend |  |  |  | <0.001 | <0.001 |

*P =0*.465 for the overall interaction between sex and self-rated health category for development of NAFLD (multivariable model).

^a^Estimated from parametric proportional hazard models. Multivariable model was adjusted for age, center, year of screening exam, smoking status, alcohol intake, physical activity, education level, total calorie intake, body mass index, sleep duration, CES-D, monthly household income, part-time job, history of hypertension, and history of cardiovascular disease.

Abbreviations: CES-D, Center for Epidemiologic Studies Depression; CI, confidence interval; HR, hazard ratio.
